# Role of Sciellin in gallbladder cancer proliferation and formation of neutrophil extracellular traps

**DOI:** 10.1038/s41419-020-03286-z

**Published:** 2021-01-06

**Authors:** Yang Li, Ruiyan Yuan, Tai Ren, Bo Yang, Huijie Miao, Liguo Liu, Yongsheng Li, Chen Cai, Yang Yang, Yunping Hu, Chengkai Jiang, Qindie Xu, Yijian Zhang, Yingbin Liu

**Affiliations:** 1 Department of General Surgery, Xinhua Hospital Affiliated to Shanghai Jiao Tong University School of Medicine, 1665 Kongjiang Road, Shanghai, 200092 China; 2 Department of Biliary-Pancreatic Surgery, Renji Hospital Affliated to Shanghai Jiao Tong University School of Medicine, Shanghai, 200092 China; 3 Shanghai Research Center of Biliary Tract Disease, 1665 Kongjiang Road, Shanghai, 200092 China; 4 Department of General Surgery, First Affiliated Hospital of Wenzhou Medical University, Baixiang Road, Wenzhou, 325000 China; 5 School of Clinical Medicine, Shanghai University of Medicine & Health Sciences, 279 Zhouzhugong Road, Shanghai, 201318 China

**Keywords:** Cancer microenvironment, Tumour biomarkers

## Abstract

Apart from primary tumor development and metastasis, cancer-associated thrombosis is the second cause of cancer death in solid tumor malignancy. However, the mechanistic insight into the development of gallbladder cancer (GBC) and cancer-associated thrombosis remains unclear. This study aimed to investigate the mechanistic role of Sciellin (SCEL) in GBC cell proliferation and the development of venous thromboembolism. The expression level of SCEL was determined by immunohistochemical staining. Roles of SCEL in gallbladder cancer cell were determined by molecular and cell biology methods. SCEL was markedly upregulated in GBC and associated with advanced TNM stages and a poor prognosis. Furthermore, SCEL interacted with EGFR and stabilized EGFR expression that activates downstream PI3K and Akt pathway, leading to cell proliferation. In addition, SCEL induces tumor cell IL-8 production that stimulates the formation of neutrophil extracellular traps (NETs), accelerating thromboembolism. In xenografts, SCEL-expressing GBCs developed larger tumors and thrombosis compared with control cells. The present results indicate that SCEL promotes GBC cell proliferation and induces NET-associated thrombosis, thus serving as a potential therapeutic target.

## Introduction

Gallbladder cancer (GBC) is the most common biliary tract malignancy and its incidence is ranked as the seventh popular one in gastrointestinal cancers^1,2^. The prognosis of patients with advanced GBC is very poor and the 5-year survival rate is only 5%, probably owing to lack of early diagnosis of this dismal disease that usually undergoes rapid progression^3–5^.

In addition to malignant transformation of cancer cells which primarily contributes to cancer death, cancer-associated thrombosis is the second leading cause of death among cancer patients^6^. Furthermore, it is emerging that the occurrence of the cancer-associated thrombosis has increased yearly worldwide^7^. Although large efforts have been significantly made to identify potential molecular mechanisms underlying venous thrombosis, the overall benefit is minimal. Mechanistically, immune cell-driven thrombosis in the vessels has recently received considerable attention, where polymorphonuclear neutrophils (PMN) release chromatin and granular proteins to form an extracellular fibrillar matrix, termed neutrophil extracellular traps (NETs)^8^. Initially in neutrophils, the nuclear histones become citrullinated by peptidylarginine-deiminase 4 (PAD4), which leads to a change in chromatin charge and facilitates chromatin decondensation. The nuclear membrane and granular membranes subsequently dissolve, thus allowing for the mixing of chromatin with granule content in the presence of the ruptured cytoplasmic membrane. A large thrombus eventually develops as a result of platelet recruitment in the NETs^9–11^. At present, the molecular regulation of neutrophil-mediated NET remains poorly understood.

Sciellin (SCEL), a precursor of the cornified envelope, was first identified in mammalian keratinizing tissues including the oral cavity, esophagus, and vagina^12^. SCEL contains 16 inexact repeats of 20 amino acid residues and an LIM domain (derived from LIN-11, Isl1, and MEC-3) that may function as a protein interaction module regulating localization of SCEL and protein assembly in the cornified envelope^13^. SCEL was enriched in stratified epithelia, where they play an important role in stress-bearing and barrier properties(8-10). Intriguingly, aberrant SCEL expression was elevated in varied malignant tumors including pancreatic cancer, colorectal cancer, and renal cancer^14–16^. However, it is largely unknown regarding the pathological role of SCEL in the development of GBC. The present study was to investigate SCEL expression in GBC and potential mechanisms underlying GBC development. We found that elevated expression of SCEL was correlated with EGFR expression and the progression of GBC. GBC cells expressing SCEL induced IL-8 secretion that in turn stimulated neutrophils extracellular traps formation that facilitate the formation of cancer-associated thrombosis. These results may shed light on identification of the novel biomarker and target for diagnosis and treatment of GBC.

## Materials and methods

### Cell culture and human sample collection

Human tumor cell lines GBC-SD were purchased from the Cell Bank of the Chinese Academy of Sciences (Shanghai, China), SGC-996 was obtained from Tongji University (Shanghai, China). The other human GBC cell lines EH-GB1 and NOZ were purchased from the Health Science Research Resources Bank (Osaka, Japan). GBC-SD, EH-GB1, and SGC-996 were cultured in DMEM, and NOZ cell line was cultured in William’s medium. All cells were cultured in their corresponding media supplemented with 10% fetal bovine serum (FBS) at 37 °C in a humidified atmosphere of 95% air and 5% CO_2_, all the cell line without mycoplasma contamination. All 49 GBC tissues were collected from patients who underwent radical surgery at the Xinhua Hospital Affiliated to Shanghai Jiao Tong University School of Medicine, Shanghai, China. We retrospectively obtained clinicopathological data from these patients, including age, sex, T stage regional lymph node status, TNM stage, and differentiation. The usage of these specimens and the patient data were approved by the Ethics Committee of the Xinhua Hospital Affiliated to Shanghai Jiao Tong University School of Medicine.

### Human peripheral blood neutrophil isolation

Whole blood was collected from patients with GBC and layered over Polymorphprep^TM^(Axis-Shield, Norway). After centrifugation at 500 × *g* for 30 min at 18–22 °C, the top band at the sample–medium interface comprised of mononuclear cells and the lower band comprised of polymorphonuclear cells were separated while pelleted erythrocytes were discarded. The polymorphonuclear fraction was then diluted with one volume of 0.45% NaCl solution or culture medium at 0.5 N to restore normal osmolality. The cell suspension was transferred to a 15 ml tube. After centrifugation at 400 × *g* for 10 min, the cells were washed with the medium followed by spinning down again and finally resuspended in culture medium.

### NETs generation

For the in vitro NET generation assay, 1 × 10^5^ neutrophil were seeded on a slide coated with poly-L-lysine and cocultured with 5 × 10^5^ tumor cells using 0.4 um transwell system for 3.5 h in RPMI1640 complete medium at 37 °C and 5% CO_2_, immunofluorescence microscopy and Western blot were used to verify NET formation.

### IHC and immunofluorescence

IHC was performed to assess SCEL and Ki-67 expression in GBC tissues. In brief, paraffin-embedded sections of GBC and cholecystitis tissue were deparaffinized and then heated in a pressure pot for 2.5 min to retrieve antigens. Thereafter, the sections were incubated with primary anti-SCEL and anti-Ki-67 antibodies (1:100, Abcam, Burlingame, CA, USA) overnight at 4 °C.Antibody binding was detected using a peroxidase–conjugated secondary antibody at 37 °C for 40 min. A DAB Substrate Kit was used to perform the chromogenic reaction. The intensity of the staining was evaluated in accordance with the following criteria: 0, negative; 1, low; 2, medium; 3, high. The extent of staining was scored as follows: 0, 0% stained; 1, 1–25% stained; 2, 26–50% stained; 3, 51–100% stained. Five random fields (20× magnification) were evaluated using a light microscope. The final scores were determined by multiplying the scores of the intensity with those of the extent and dividing the samples into four grades: 0, negative (-); 1–2, low staining (+); 3–5, medium staining (++); 6–9, high staining (+++)^17,18^. Immunofluorescence staining was performed to detect the neutrophils. In brief, NETs were added on 13 mm glass coverslips (Fisher Scientific) and allowed to adhere overnight at 4 °C on coverslips followed by culture in the presence of anti-citH3 (1:100, NOVUS, USA) and NE (1:100, NOVUS) antibody at 4 °C overnight. Thereafter, cells were incubated with fluorescent dye-labeled secondary antibodies at room temperature for 1 h. The cells were again incubated with anti-fade DAPI solution (1:1000), and images were captured with a confocal fluorescence microscope.

### Establishment of an in vivo tumor model

All animal studies were approved by Xinhua Hospital Ethics Committee Affiliated to Shanghai Jiao tong University School of Medicine, and 4-week-old female BALB/c nude mice were maintained in a barrier facility on high-efficiency particulate air-filtered racks. Tumor cells were harvested via trypsinization, washed in PBS, and resuspended at 1 × 10^7^ cells/ml in Matrigel. A total of 1 × 10^6^ cells were subcutaneously injected into each mouse to develop tumors as previously described^3^. Tumor size was measured weekly.

### Thrombosis model

After BALB/c nude mice had subcutaneously received tumor cells for 8 weeks, the inferior vena cava (IVC) was exposed, carefully separated from the aorta, and fully ligated with 5-0 silk suture below the left renal vein. Any side branches close to the IVC ligation site were also ligated, and the back branches were cauterized. Clots were harvested at 48 h and weighed, and then clots were performed HE staining.

### Neutrophil count analysis

A Hemavet HV950FS (Drew Scientific, Miami Lakes, FL, USA) was used to count neutrophils in the blood.

### Quantification of autonomous NET formation in patients with GBC and mice model

To quantify NET formation in patients with GBC and mice model, we measured the amount of circulating myeloperoxidase (MPO)–DNA complex, a well-established marker of NET formation, using a modified capture ELISA technique as previously described^19,20^.

### RT-PCR

The total RNA of the cells was extracted with TRIzol (Invitrogen) in accordance with the manufacturer’s instructions. Thereafter, the mRNA was reverse-transcribed to single-stranded cDNAs using a reverse-transcription PCR (RT-PCR) system (TaKaRa). The primers were listed in Supplementary Table 2. Thereafter, real-time fluorescent quantitative PCR or semi-quantitative PCR was performed to analyze the gene expression levels.

### Western blotting

Whole-cell extracts were prepared by lysing the cells with RIPA lysis buffer supplemented with a proteinase inhibitor cocktail (Sigma). In total, 30 ug protein lysate was separated via SDS–PAGE and then the target proteins were detected via Western blot analysis with the following antibodies: anti-SCEL, anti-PI3Kα110, anti-EGFR, anti-P-AKT(ser473), anti-pan-AKT, anti-FLAG, and anti-β-actin (Supplementary Table 1).

### CCK8 assay

CCK8 assays were performed in accordance with the manufacturer’s instructions. In brief, 1000 cells in 100 ul culture were added into each well of a 96-well plate. The plate was incubated for a period (0, 1, 2, 3, 4, and 5 days) in an incubator and then 10 ul of CCK-8 solution was added to each well of the plate. The plate was again incubated for 2 h in the incubator and the absorbance was measured at 450 nm using a microplate reader. To perform colony-formation assay, GBC cells were seeded on six-well plates (500 cells/well). After culturing for 2 weeks, colonies were fixed with 4% paraformaldehyde for 30 min and stained with 0.1% Crystal Violet (Beyotime) for 20 min. Thereafter, stained colonies were photographed and the numbers of colonies (>50 cells/colony) were counted after rinsing twice with PBS. Three independent experiments were performed.

### Plasmid construction and stable cell line establishment

The complete coding region of human SCEL (NM_003843.4) was cloned into pCMV Puro vector. Lentiviruses were produced in 293 T cells for the stable transfection of the cell lines, in accordance with the manufacturer’s instructions, and an empty vector was transfected into cells as a control. In total, 1 × 10^5^ tumor cells in 2 ml medium with 8 μg/ml polybrene were infected with 1 ml lentivirus supernatant. After 48 h, cultures were supplemented with puromycin (4 μg/ml) for 3 days for drug-resistance selection.

### Co-immunoprecipitation assays (Co-IP)

EH-CB1 cells were transfected with different expression plasmids and lysed. Cell lysates were incubated with anti-FLAG antibody conjugated beads at 4 °C overnight. The beads were washed with lysis buffer three times followed by Western blotting.

### ELISA

GBC cell lines (2 × 10^5^ cells) were implanted in 6-well plates and cultured for 72 h, and the conditioned medium was collected after centrifugation at 700 × *g* for 5 min at 4 °C. IL8 protein was quantified using IL8 kit (BOSTER Systems) according to the manufacturer’s instructions. The same culture medium was used as a control.

### Statistical analysis

Statistical analyses were performed using the IBM SPSS Statistics Program. Each experiment was performed in triplicate, and the values are presented as the mean ± SD, unless otherwise stated. The variance between the groups was statistically compared. Student’s unpaired *t* test was used to compare the mean values. Kaplan–Meier curves were analyzed for relevant variables. The log-rank test was used to analyze the differences in survival times among the patient subgroups. All probability values had a statistical power of 90%, and a 2-sided level of 5%. A *P*-value <0.05 was considered statistically significant.

## Result

### SCEL is overexpressed in GBC tissues

**Fig. 1:**
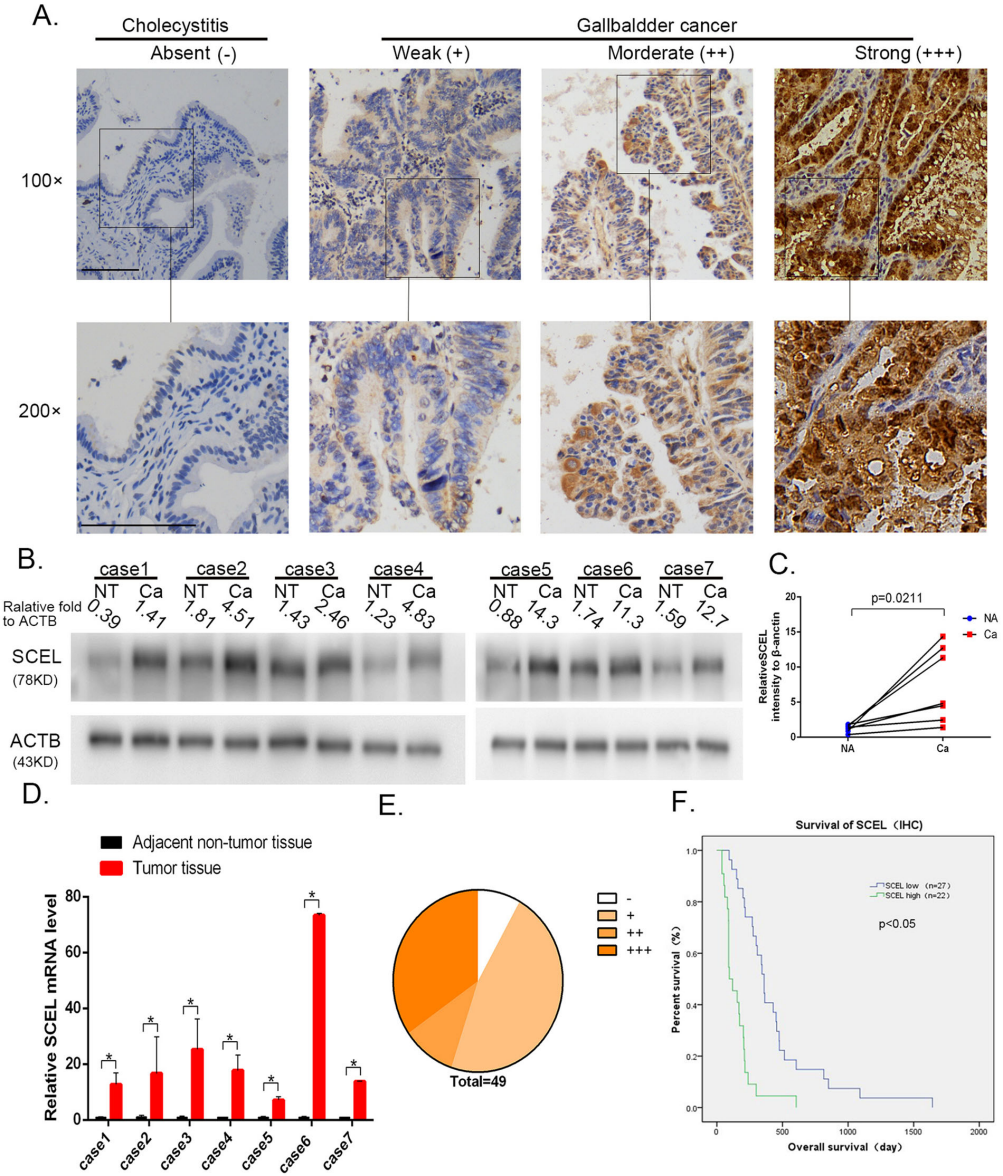
The expression and clinical significance of SCEL in GBC tissues. **A** Representative image was shown for absent, weak, moderate, and strong expression of SCEL in IHC staining of cholecystitis and GBC tissue. Squared boxes in the top images were amplified at the bottom. Bar: 100 um. **B**–**D** Seven pairs of fresh GBC tumor tissues and NT tissues were subjected to Western blotting analysis (**B**, **C**) and RT-PCR (**D**) to compare the expression levels of SCEL. *P < 0.05. **E** The distribution of IHC results in 49 GBC tissues. **F** Kaplan–Meier analysis of overall survival of the 49 GBC patients according to different SCEL levels.

To investigate the expression levels of SCEL in GBC, we performed IHC staining of GBC tumor tissues and the cholecystitis tissue as controls. As shown in Fig. 1A, SCEL was overexpressed in tumor tissues and rarely expressed in the cholecystitis count parts. Subsequently, SCEL expression in 7 pairs of fresh GBC tissues and the tumor-adjacent normal tissues were analyzed using Western blot analysis (Fig. 1B, C) and RT-PCR (Fig. 1D). The results indicated that SCEL was overexpressed in GBC tumor tissues (*P* = 0.0211; Fig. 1C).To validate this elevated level in GBC and clinical relevance of SCEL with the development of GBC, we extended our study to enroll 49 human GBC specimens and analyzed expression levels and relationship of SCEL with clinical outcomes (Fig. 1E, Table 1). SCEL upregulation was positively correlated with the T stage (*P* = 0.037) and pTNM stage (*P* = 0.047). The overall survival times were significantly longer in GBC patients with low levels of SCEL than in those with high levels of SCEL (Fig. 1F). All the data suggest SCEL was elevated in GBC and may be associated with poor prognosis.

**Table 1:** Association between SCEL expression and clinicopathological features of patients with gallbladder cancer.

Variables	SCEL expression		Total (*n* = 49)	*X*^2^	*P* value
	Low (*n* = 19)	High (*n* = 53)			
*Age*				1.538	0.215
°<65	12	6	18		
°≥65	15	16	31		
*Gender*				0.328	0.567
°Male	12	8	20		
°Female	15	14	29		
*T staging*				4.331	0.037*
°T1 + T2	14	5	19		
°T3 + T4	13	17	30		
*Nodal staging*				*0.678*	0.407
°N0	13	8	21		
°N1	14	14	28		
*Differentiation*				4.067	0.103
°Well	9	9	18		
°Moderate	11	12	23		
°Poor	7	1	8		
*TNM*				3.96	0.047*
°I-IIIA	15	6	21		
°IIIB/IV	12	16	28		

Statistical analyses were performed with the Chi-square test. *P < 0.05 was considered statistically significant.

### SCEL regulates tumor cell growth in vitro and vivo

**Fig. 2:**
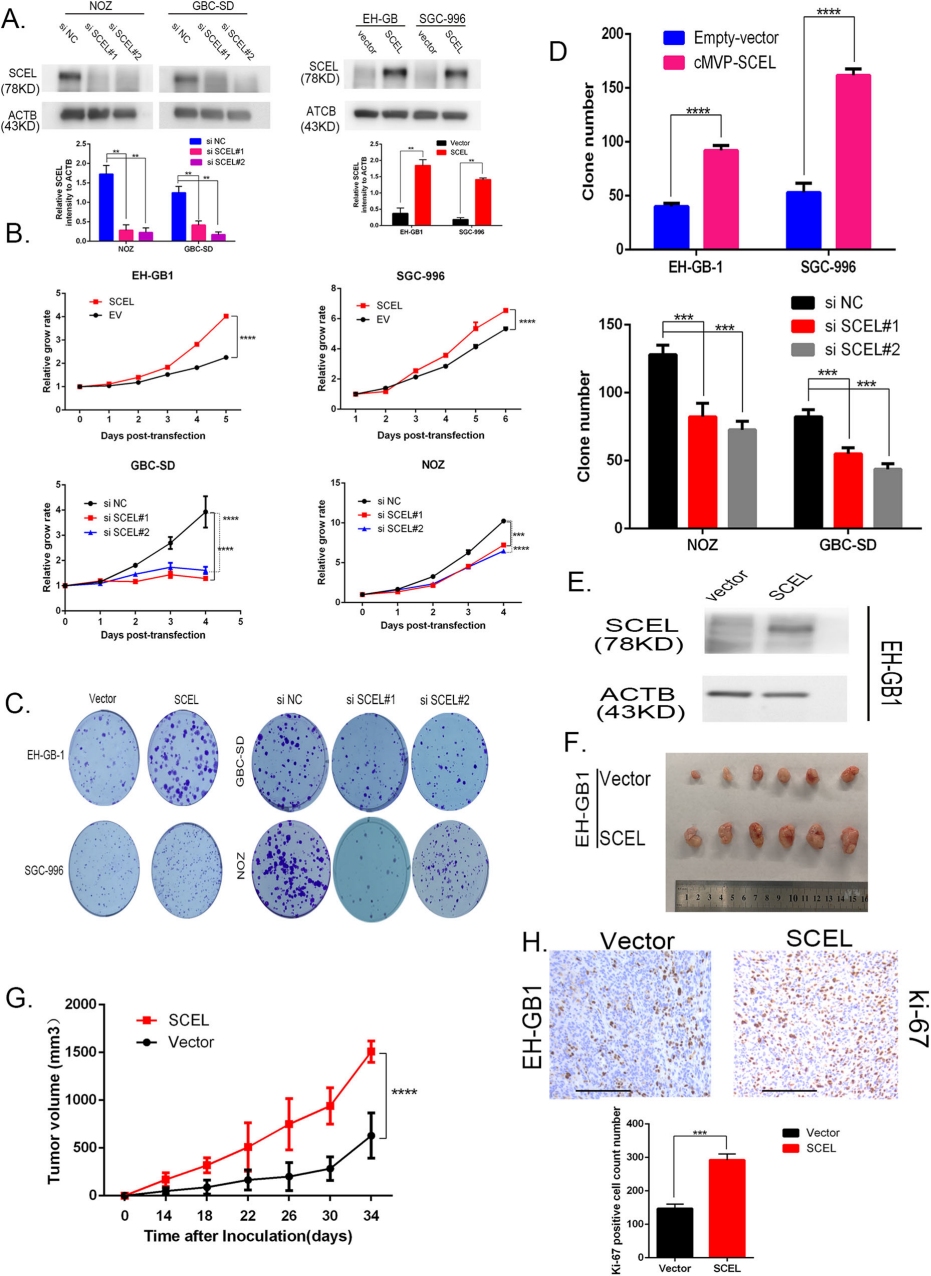
SCEL promotes GBC cell proliferation and tumor growth. **A** The indicated GBC cells were transfected with small interfering RNA or SCEL gene plasmid to knock down or overexpress SCEL, respectively. Western blotting was performed to verify these cell lines, grayscale statistics at the bottom of Western blot band. **B**, **C** The colony formation was assessed in GBC cells and statistical significance was analyzed based on the numbers of colonies. **D** The proliferation of treated GBC cells was measured by CCK8 assay. **E** Expression levels of SCEL were examined in EH-GB-1 cells transfected with Lv-EV and Lv-SCEL. **F** Above tumor cells were subcutaneously transplanted into nude mice to develop tumors (*n* = 6 for each group). **G** The tumor size was measured twice a week, and the tumor growth curve was generated based on the mean tumor volume. **H** The expression of Ki67 was detected, statistical analysis (unpaired test) of Ki-67 positive cell count number under the IHC picture; Bar: 100 um. Unpaired *t* tests were used in **C**, **D**, **G**, and **H**; data are shown as the mean value ± SD; *P < 0.05.

To explore the pathologic activity of SCEL in GBC cells, SCEL expression levels were determined in five GBC cell lines (Supplementary Fig. S1). We used two siRNA1 and siRNA2 to silence SCEL in two GBC cell lines NOZ and GBC-SD cell that harbored relatively higher expression levels of SCEL, while EH-GB1 and SGC-996 cells with lower expression were engineered to ectopically express SCEL. The resultant expression levels of individual cell lines were confirmed through Western blot analysis (Fig. 2A). Cell proliferation with CCK8 assay showed that EH-GB1 and SGC-996 cells expressing SCEL induced cell growth compared with control cells, whereas NOZ and GBC-SD cells expressing SCEL siRNA1 and siRNA2 decreased cell proliferation (Fig. 2B). Likewise, colony-formation assays resulted in the same pattern as cell proliferation (Fig. 2C, D).

To evaluate the effects on cell growth in vivo, we established EH-GB1 cell line stably expressing SCEL and subcutaneously transplanted them into nude mice (Fig. 2E). Overexpression of SCEL in EH-GB1 cells led to large tumors than those developed from control cells (Fig. 2F, G). Consistent with this data, IHC revealed increased Ki-67-positive cells in the tumors overexpressing SCEL compared with control tumors (Fig. 2H), suggesting that SCEL promotes tumor cell growth both in vitro and in vivo.

### SCEL promotes gallbladder cancer growth by stablizing EGFR protein expression

**Fig. 3:**
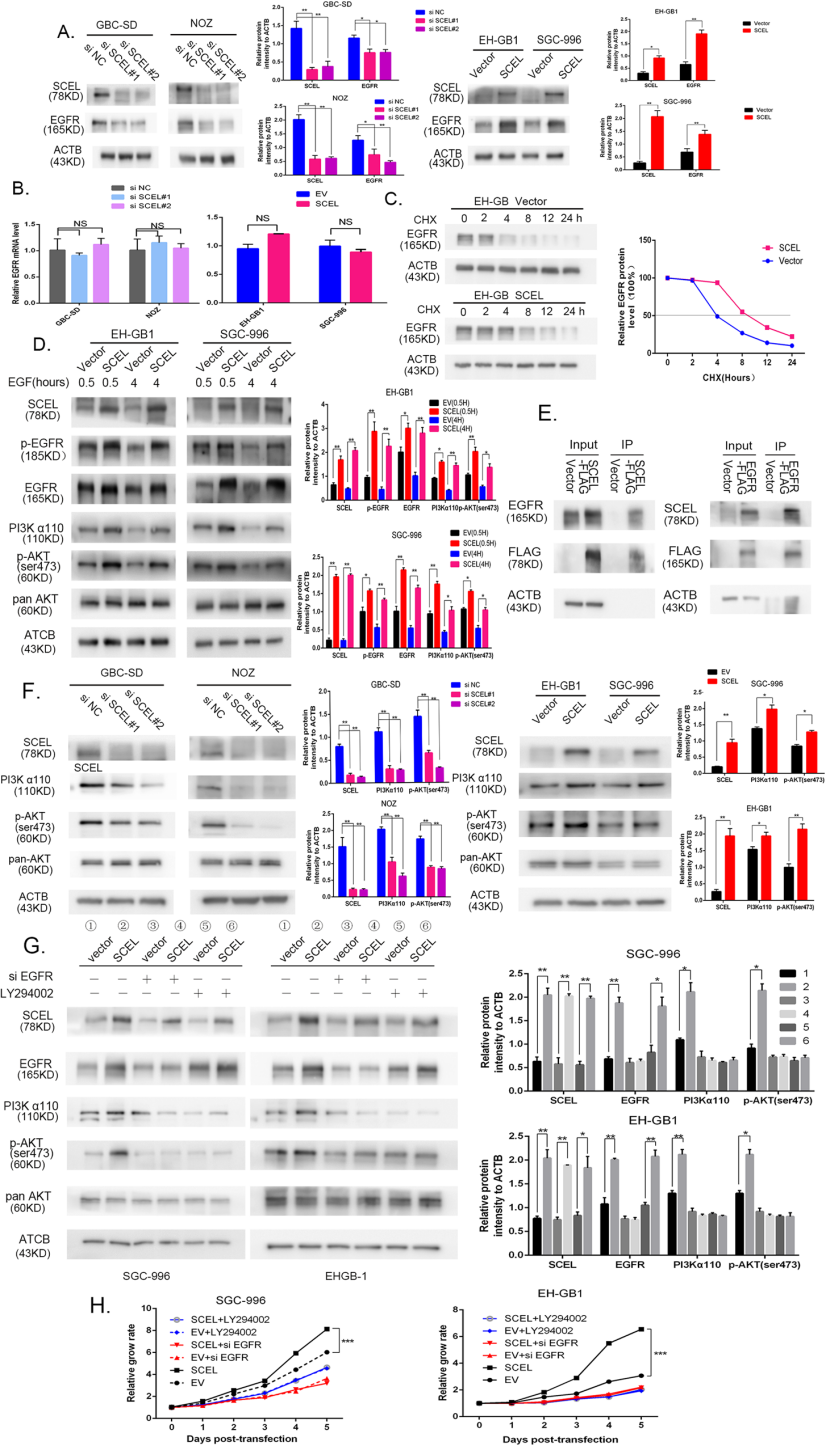
SCEL promotes gallbladder cancer growth by stablizing EGFR protein expression. **A**, **B** GBC cells with SCEL upregulation or downregulation were subjected to Western blotting (**A**) and RT-PCR (**B**) to detect the expression level of EGFR, grayscale statistics on the right side. **C**, EH-GB1 cells were transfected with EV and SCEL plasmid. After 12 h, cells were treated with 100 μM cycloheximide (CHX) for time periods as indicated. Western blotting analysis was conducted with EGFR and ACTB, representative immunoblots and the ratio of the indicated protein to ACTB are presented. **D** Control and SCEL overexpression plasmid- transfected (2 days) cells were serum-starved and stimulated with EGF for indicated times. Cell lysates were prepared and analyzed by immunoblotting using indicated antibodies, grayscale statistics on the right side. **E** Co-immunoprecipitation and Western blot analysis of SCEL and EGFR proteins from EH-GB1 cell line after transfected with SCEL-3FLAG or EGFR-3FLAG plasmid. **F** The indicated GBC cells subjected to Western blotting to detect the level of PI3Kα110, p-AKT(ser473) pan AKT, grayscale statistics on the right side. **G** The indicated GBC cells were incubated with or without EGFR siRNA20 μM LY294002 and then subjected to Western blotting to detect the expression level of EGFR, PI3Kα110, p-AKT(ser473), pan AKT. The incubation times is as follows: LY294002, 12 h, grayscale statistics on the right side. **H** GBC cells were transfected EV or SCEL plasmid and treated EGFR siRNA or LY294002 and CCK8 assay was perfromed to determine its proliferation. *p < 0.05; **p < 0.001, ***p < 0.001.

As our previous study that revealed EGFR is a master regulator in GBC tumor cell growth^21^, we were particularly interested in possible regulation of SCEL in EGFR gene expression. To test this possibility, GBC-SD and NOZ cells expressing SCEL siRNA1 and siRNA2 were examined for EGFR expression. As shown in Fig. 3A, EGFR expression was decreased in both SCEL siRNA1 and siRNA2-expressing GBC-SD and NOZ cells relative to controls. In contrast, overexpression of SCEL in EH-GB1 and SGC-996 cells induced EGFR expression. Immunofluorescence analysis on GBC-SD cells expressing GBC siRNA displayed the same effect on cell surface EGFR (Supplementary Fig. S2), implicating that SCEL regulates EGFR. Next, to explore if SCEL upregulates EGFR expression at transcriptional and/or post-transcriptional levels, we first examined EGFR mRNA levels. SCEL overexpression or knockdown did not alter EGFR mRNA levels (Fig. 3B). Then, we used cycloheximide to inhibit protein synthesis to determine if SCEL has the ability to stabilize protein expression. As shown in Fig. 3C, when GBC cells overexpressing SCEL were treated with CHX, the degradation of EGFR in 24 h was slower than it was in control cells, supporting our hypothesis that SCEL induces EGFR expression at the post-transcriptional level. Consistently, EGFR activation and signaling were increased in intensity and duration in SCEL overexpression of plasmid-transfected cells (Fig. 3D). Furthermore, immunoprecipitation of SCEL followed by immunoblotting with an EGFR antibody showed physical association of SCEL with EGFR, identical to the reciprocal approach (Fig. 3E), indicating that SCEL may mediate EGFR degradation, inhibiting its proteasomal degradation.

As EGFR downstream pathway through PI3K/Akt1, we evaluated if this pathway is altered by SCEL. Silence of SCEL in GBC-SD and NOZ cells suppressed activated PI3Kα110 and phosphorylated Akt1 at serine 473, whereas overexpression of SCEL in EH-GB1 and SGC-996 cells increased these kinase levels (Fig. 3F). When an EGFR siRNA was introduced to EH-GB1 and SGC-996 cells overexpressing SCEL, siRNA completely abrogated SCEL-induced EGFR, PI3Kα110 upregulation and AKT1 phosphorylation (Fig. 3G). Moreover, a PI3K inhibitor (LY294002) that failed to block EGFR expression also completely inhibited PI3Kα110 and AKT1 phosphorylation.

Analogous to the EGFR signaling blockade, both EGFR and PI3Kα siRNA or inhibitors attenuated cell proliferation induced by SCEL (Fig. 3H), suggesting that SCEL acts as a tumor-promoting factor by activating EGFR-PI3K-Akt signaling.

As Fig. 3D shows SCEL not only influence EGFR expression, but activate EGFR -Y-1068, so we explored whether SCEL promotes proliferation by activating p-EGFR. In Supplementary Fig. S3A, the data showed that SCEL overexpression enhanced the phosphorylation of EGFR, AKT, EGFR inhibitor erlotinib completely abrogated the SCEL-induced phosphorylation EGFR, AKT, as well as SCEL-induced PI3Kα110 expression. Moreover, the LY294002 also completely inhibited the SCEL-induced phosphorylation AKT and PI3K. In vitro, erlotinib and LY294002 showed similar growth-inhibiting effects as well as SCEL knockdown (Supplementary Fig. S3B).

### SCEL influences the neutrophil extracellular traps formation

**Fig. 4:**
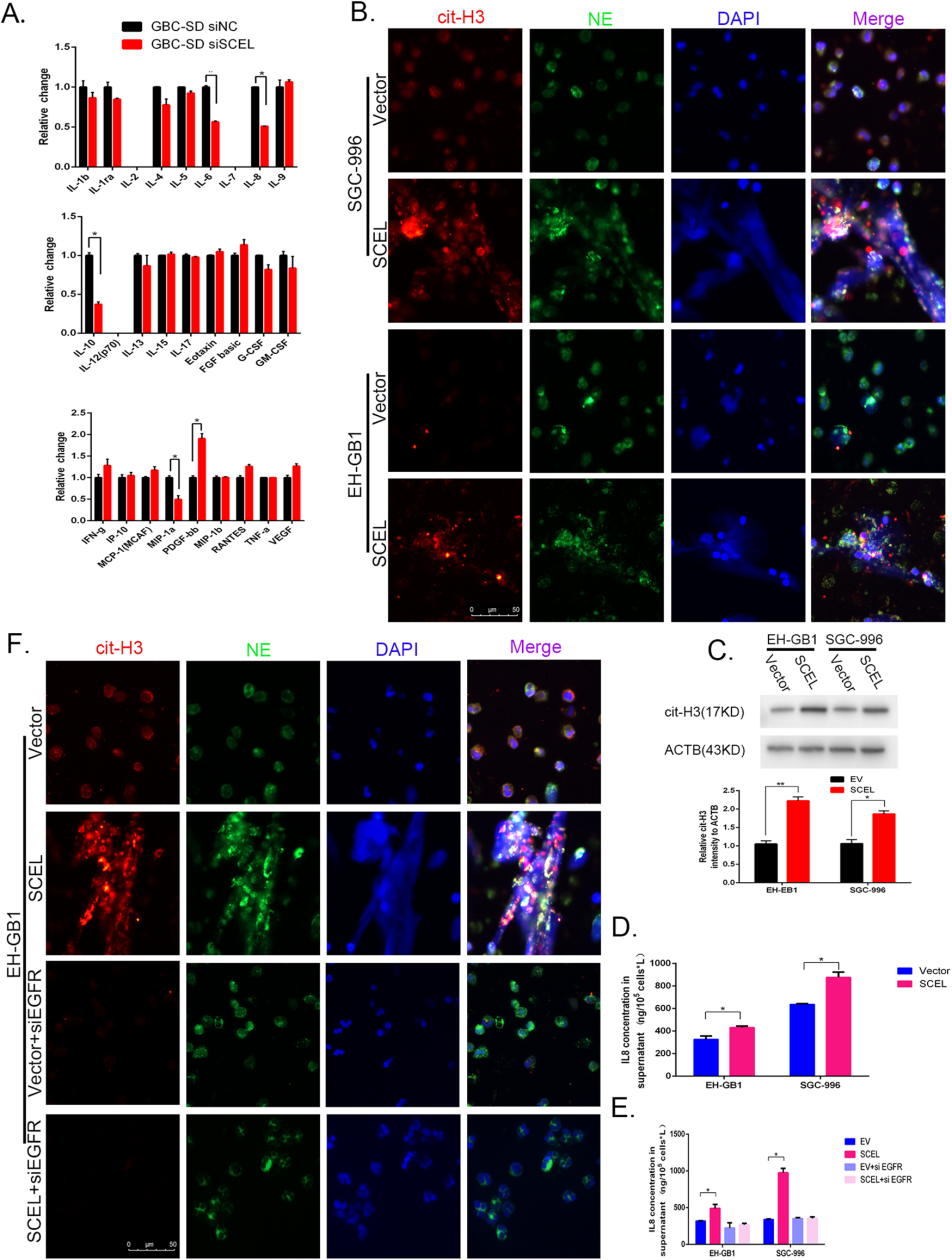
SCEL influence the neutrophil extracellular traps formation. **A** The indicated cells conditioned medium were performed liquid suspension array. **B** Neutrophils were isolated from peripheral blood coculture with the indicated GBC cells for 3.5 h and then representative immunofluorescent staining photographs of cit-histone3 (red), NE (green) and DAPI (blue) are shown by fluorescence microscope. **C** Cit-histone H3 protein levels were determined by Western blot, grayscale statistics at the bottom of Western blot band. **D** ELISA were performed to verify the regulatory effects of SCEL on IL8 in indicated cell lines. **E**, **F** The indicated GBC cell lines were incubated with or without EGFR siRNA and then subjected to ELISA array to detect the IL8 concentration (**E**) or cocultured with neutrophil to identity the neutrophil extracellular traps formation (**F**). *p < 0.05, **p < 0.001, ***p < 0.001.

To interrogate if SCEL overexpression in tumor cells can influence adjacent stromal cells that alter TME favorable for tumor progression, we used a liquid suspension array method and found that IL-6, IL-8, IL10, and MIP-1a were markedly downregulated when SCEL was knocked down in GBC-SD (Fig. 4A). As potent factors are involved in inflammation and immune reaction, we were interested in effects of IL6,IL8, and MIP-1a on neutrophils that participate in the development of NET. In contrast with IL-6 and MIP-1a, IL8 significantly induced NET formation that was characterized with specific markers of citrullinated-histone H3 (cit-H3) and neutrophil elastase (NE) in neutrophils (Supplementary Fig. S4)^8^ consistent with early report^22^. Then we used a co-culture system as neutrophils were grown at the bottom chamber and EH-GB1-Vector or EH-GB1-SCEL cells at the top chamber of a transwell 3.5 h later, NETs in neutrophils were detected (Fig. 4B). In line with these phenotypes, EH-GB1 and SGC-996 cells expressing SCEL induced neutrophil to express high levels of citrullinated-histone H3(cit-H3) (Fig. 4C). Furthermore, ELISA revealed that IL-8 was upregulated in SCEL-overexpressing GBC cells (Fig. 4D). It was reported that EGFR influences IL8 production^23^, we then tested if induced IL-8 and subsequent NETs in SCEL-expressing cells are dependent on EGFR. To test this, we treated the cells with EGFR siRNA and found that it completely abrogated SCEL-induced IL-8 production in both EH-GB1 and SGC996 (Fig. 4E). Moreover, EGFR siRNA and IL-8 neutralization completely inhibited GBC-SCEL-induced NET formation in EH-B1 cells (Fig. 4F, Supplementary Fig. S5). These results suggest that SCEL influences NETs formation via EGFR/IL8 signaling.

### SCEL influences the venous thromboembolism

**Fig. 5:**
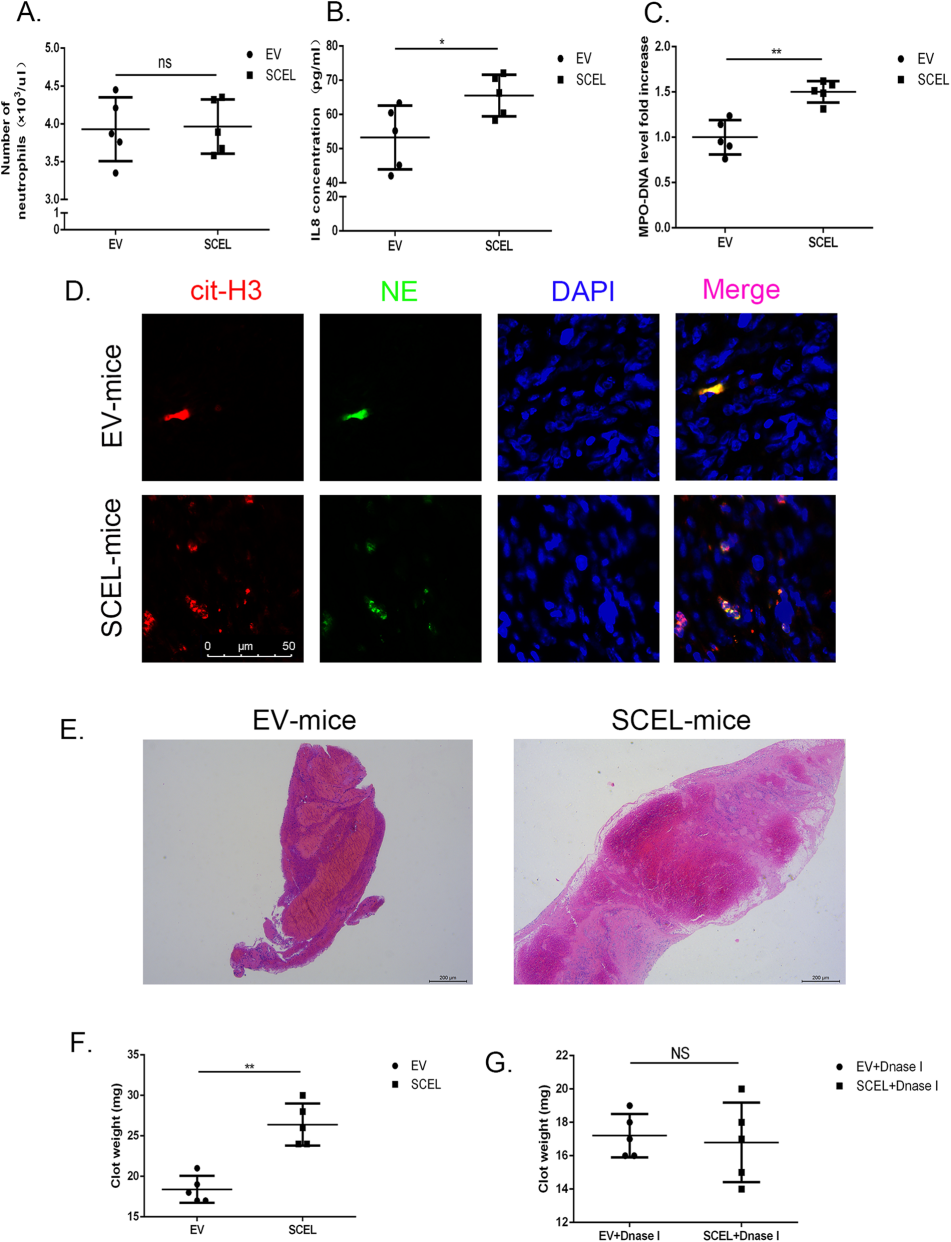
SCEL influences the venous thromboembolism formation in tumor-bearing mice. The indicated GBC cells were subcutaneously transplanted into nude mice to develop tumors. Tumors were grown for 8 weeks. Venous thrombosis in mice was induced by ligation of the inferior vena cava (IVC). **A** The numbers of neutrophils in blood. **B**–**C** Level of IL8 concentration (**B**) and MPO–DNA (**C**) in the plasma from the different groups of mice. **D** Detection of NETs by immunofluorescence in tumor. **E** Clots were embedded in paraffin, sectioned longitudinally, and stained with hematoxylin and eosin. **F** Clots were harvested at 48 h after IVC ligation and weighed. **G** The indicated subcutaneously tumor mice received DNase I (50 Unit/mouse) 1 h before inferior vena cava (IVC) stasis and clot were collected at 48 h and weighed. *p < 0.05, **p < 0.001, ***p < 0.001.

NETs are strongly thrombogenic, as platelets are sequestered into NETs, thus initiating thrombus formation^9–11^.To visualize the effects of SCEL-induced NET on the formation of venous thrombosis, we employed a standard venous thrombosis model by ligation of the inferior vena cava in tumor mice that had subcutaneously received tumor cells for 8 weeks^24^. First, we found there was a significant increase IL8 concentration and circulating myeloperoxidase (MPO)–DNA complex, a well-established marker of NETs formation, in the plasma of SCEL overexpressed group than in control, but no neutrophil number difference between two groups (Fig. 5A-C), the same result can also be observed in the gallbladder cancer patients sample (Supplementary Fig. S6). Furthermore we also found more NET numbers in SCEL overexpressed tumors bearing EH-GB1 cells compared with control tumors (Fig. 5D). As shown in Fig. 5E, the clots in the SCEL group were larger than those in the EV group. Clot weight significantly increased at 48 h after IVC ligation in SCEL overexpressed tumor-bearing mice (Fig. 5F). On using DNase I for EV and SCEL tumor mice, DNase I significantly reduced the clot sizes in these groups, and no difference was observed between these two groups (Fig. 5G), indicating that SCEL influences the formation of venous thromboembolism by NETs.

## Discussion

Tumor metastasis and tumor-associated thrombosis in solid tumor development are the two key events of tumor malignant transformation that lead to cancer death^6^. Albeit there is an ample body of evidence documented to regulate cancer invasiveness at the cellular and molecular levels, little is known regarding the molecular mechanisms that regulate tumor-associated thrombosis. It is emerging that tumor-associated neutrophil-mediated NETs play a central role in the venous thrombosis during tumor progression. In the current study, SCEL was found to stimulate IL-8 production that promotes neutrophil-mediated NETs in GBC. Indeed, SCEL was significantly upregulated in GBC. SCEL upregulation was correlated with T staging, TNM stages, and poor survival of tumor patients. Therefore, SCEL may serve as a diagnostic biomarker and a therapeutic target in GBC treatment.

EGFR is a tyrosine kinase receptor that is overexpressed in many tumor types, including GBC^25–27^. Overexpression or activating mutation of the EGFR promotes tumor proliferation, angiogenesis, and metastasis and so on. Overexpression or activating mutations in the kinase domain of EGFR increase the kinase activity of EGFR, leading to the hyperactivation of downstream pro-survival genes, and consequently confer oncogenic properties on EGFR, endocytic trafficking and degradation are main EGFR regulator system^28^, here we found SCEL promotes tumor cell growth via stabilizing EGFR expression in the cells, rather than transcriptional regulation. In addition, upregulated EGFR activates PI3K and downstream Akt, thus driving cell proliferation. Although SCEL is associated with EGFR, it remains to be established how SCEL protects EGFR from degradation and where this degradation occurs. We interestingly found that EGFR inhibitor suppressed EGFR expression and corresponding tumor cell growth, but its inhibition on cell proliferation was even lower than the control level, regardless of decreased EGFR expression comparable to control levels. It can be not excluded at the present that other cell growth-associated proteins may be inhibited by impaired EGFR signaling or EGFR inhibitor itself.

Cancer is related to arteries as well as vein thrombosis^29,30^. The mechanism by which tumors cause thrombus formation is not fully understood, and several possible mechanisms have been proposed^31–33^. Cancer is closely related to inflammatory response^34^. Current studies have shown that NETs play role in tumor progression and metastasis^35–43^, a growing number evidence suggests tumor cells, tumor-educated platelets, tumor- or host-secreted cytokines, such as granulocyte colony-stimulating factor (G-CSF), and IL-8 induce NETs formation in cancer^44,45^. In this study, we found that SCEL stimulates multiple cytokine expression in tumor cells, in which IL-8 was found to promote neutrophils to develop NETs in vitro, consistent with previous reports that IL-8 is an essential factor in the formation of NETs^46^. Together, these data suggest that SCEL is associated with systemic changes that prime neutrophils to release NETs in gallbladder cancer. In an in vivo model, we found that GSCs expressing SCEL augment venous thrombosis with higher level of IL8 concentration and MPO–DNA complexes in plasma. The same result can be seen in the GBC patient sample with SCEL high expression. It warrants substantial investigation on the potential role of IL-8 in the development of NETs and thrombosis during tumor growth; however there are a myriad of mechanisms that cause venous thromboembolism in gallbladder cancer patients. This research may be useful for identifying other pathways that may contribute to VTE in gallbladder cancer patients. Furthermore, we have not observed obvious neutrophilia in our animal model; this result differs from recent studies employing murine models of breast cancer^47^, and this may be because of heterogeneity of cancer.

Several potential limitations of this study should be noted. First, we only identify SCEL can physically associate with EGFR, and propose promising mechanism that SCEL mediated EGFR stabilization and activation by binding with EGFR, it is still needed to verify our findings. Second, extracellar traps not only can be seen in the neutrophil but macrophage and some other cells^19,48^, so MPO–DNA complexes in the plasma cannot exclusively represent neutrophil extracellar traps. Further studies are needed to verify this. Our study is the first identify that SCEL can promote gallbladder cancer cell proliferation by EGFR/PI3K/AKT pathway, and SCEL can influence neutrophil extracellular traps formation through EGFR/IL8 axis.

## Supplementary information

Suppl. Fig. S1

Suppl. Fig. S2

Suppl.Fig. S3

Suppl.Fig. S5

Suppl.Fig. S4

Suppl.Fig. S6

suppl.figure legend

suppl.Table1

suppl.Table2

## Data Availability

The dataset supporting the conclusions of this article is included within the article.
