# Supplementary figures and images for: Role of Sciellin in gallbladder cancer proliferation and formation of neutrophil extracellular traps
Source: Cell Death Dis. 2021 Jan 6;12(1):30. doi: 10.1038/s41419-020-03286-z (PMC7791032; doi:10.1038/s41419-020-03286-z)

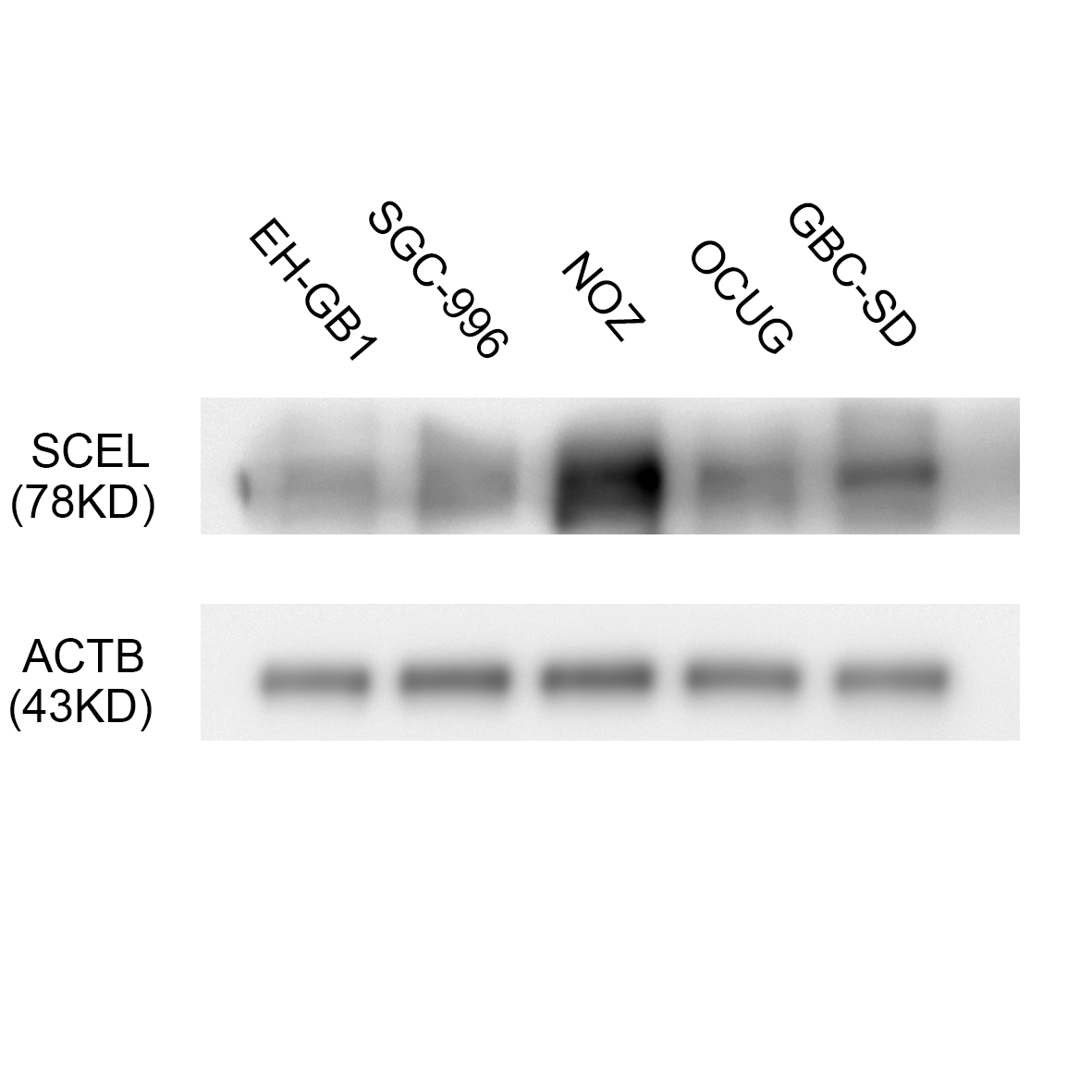

Supplement: Supplementary file 1 — Suppl. Fig. S1 [file 41419_2020_3286_MOESM1_ESM.tif]

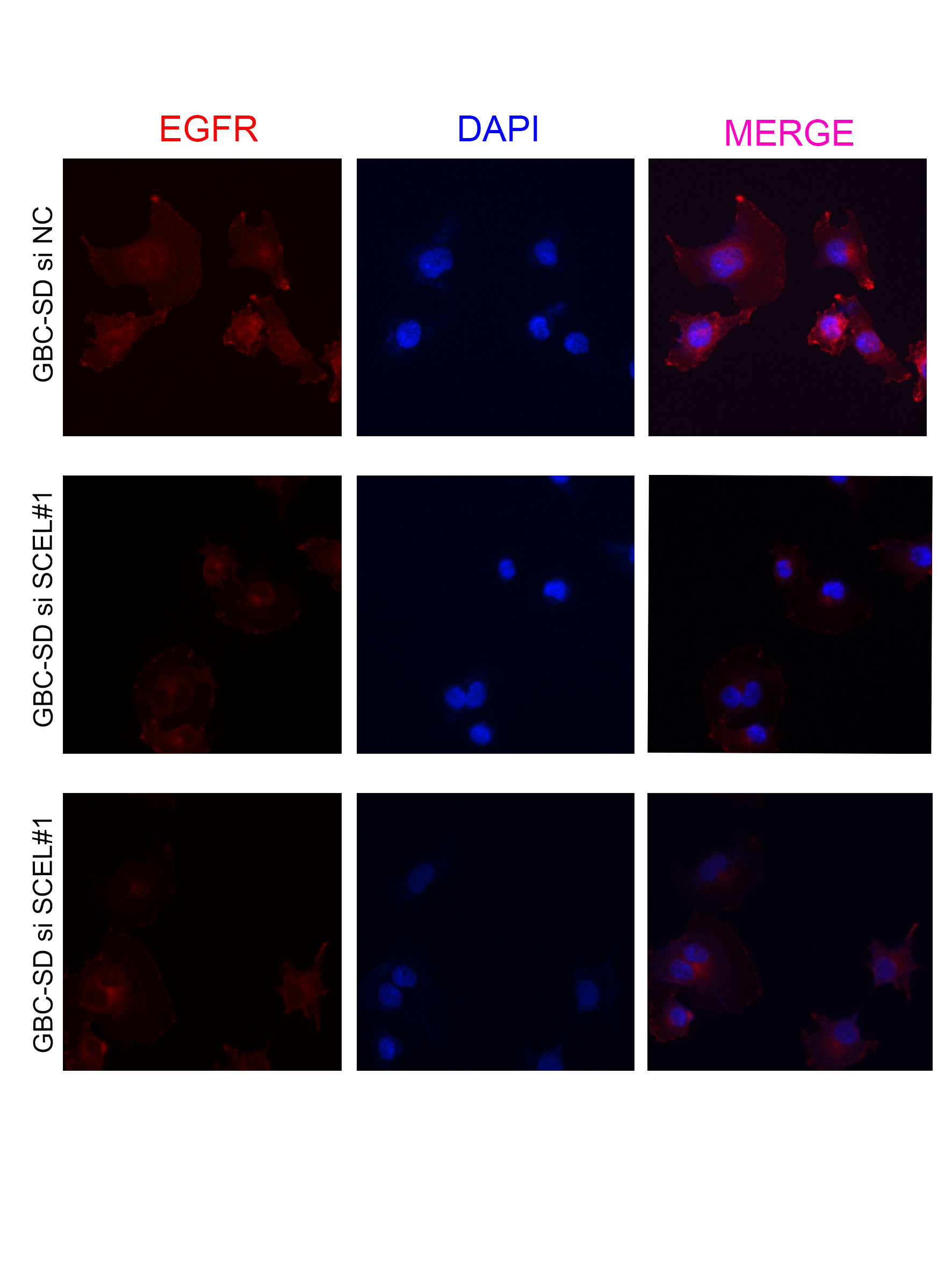

Supplement: Supplementary file 2 — Suppl. Fig. S2 [file 41419_2020_3286_MOESM2_ESM.tif]

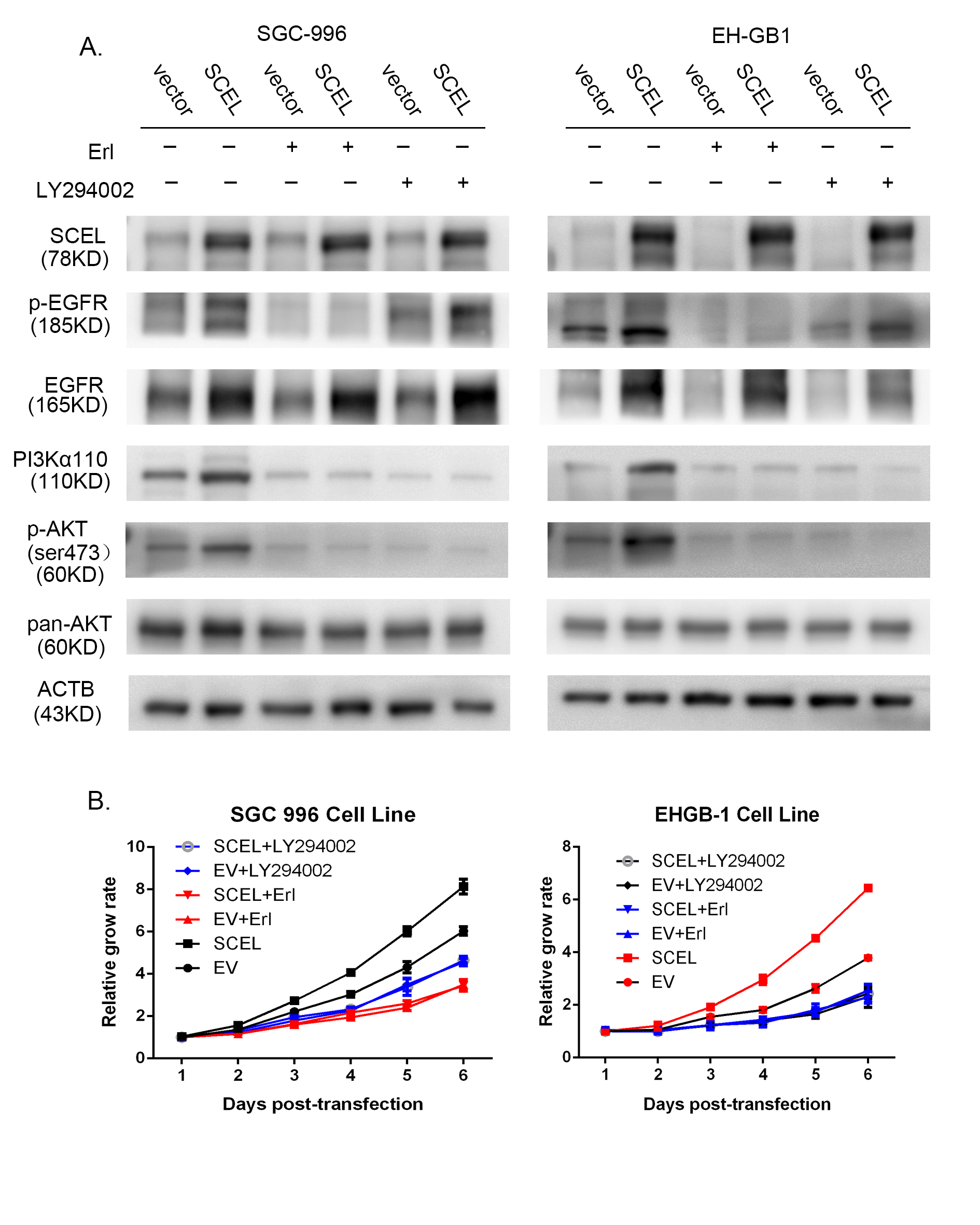

Supplement: Supplementary file 3 — Suppl.Fig. S3 [file 41419_2020_3286_MOESM3_ESM.tif]

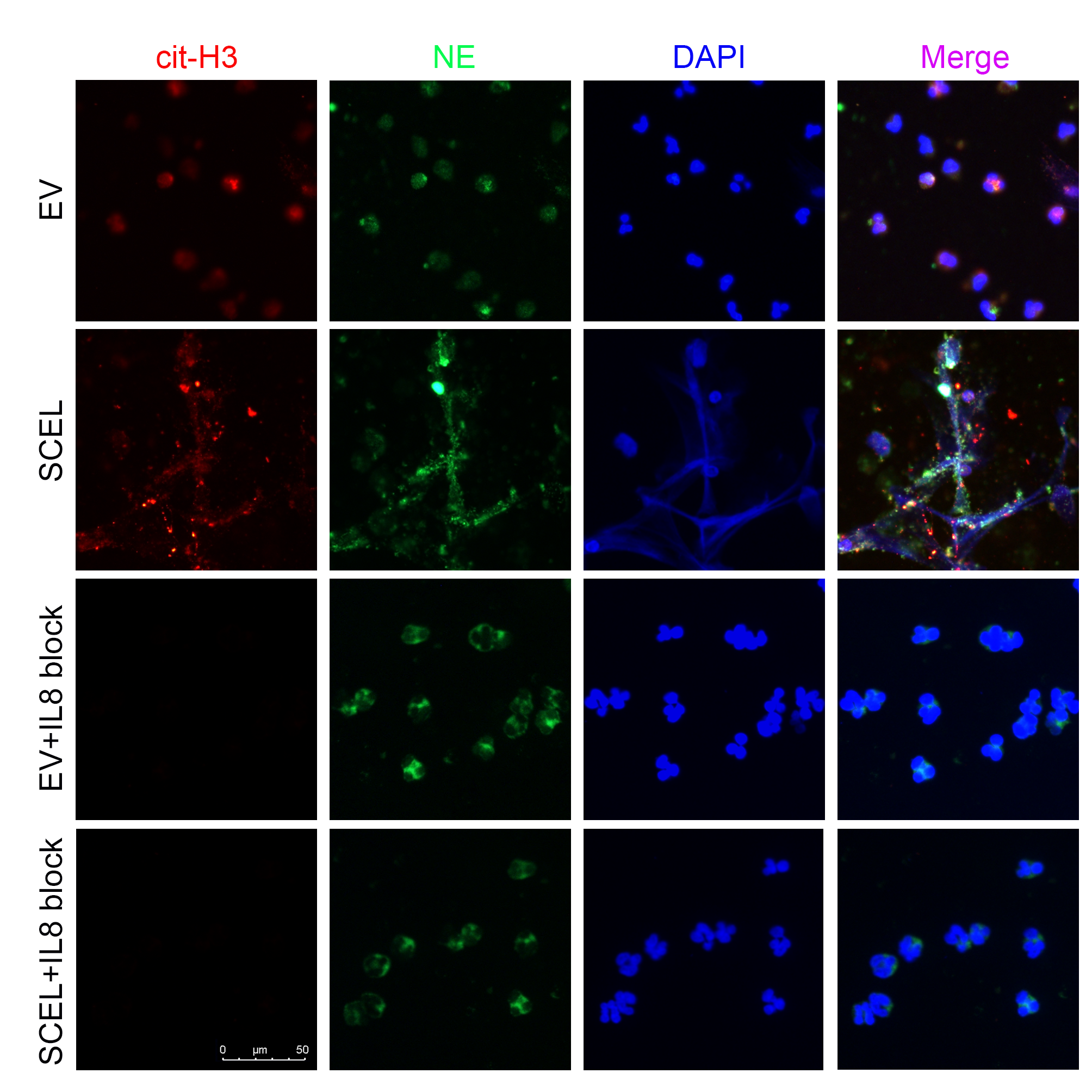

Supplement: Supplementary file 4 — Suppl.Fig. S5 [file 41419_2020_3286_MOESM4_ESM.tif]

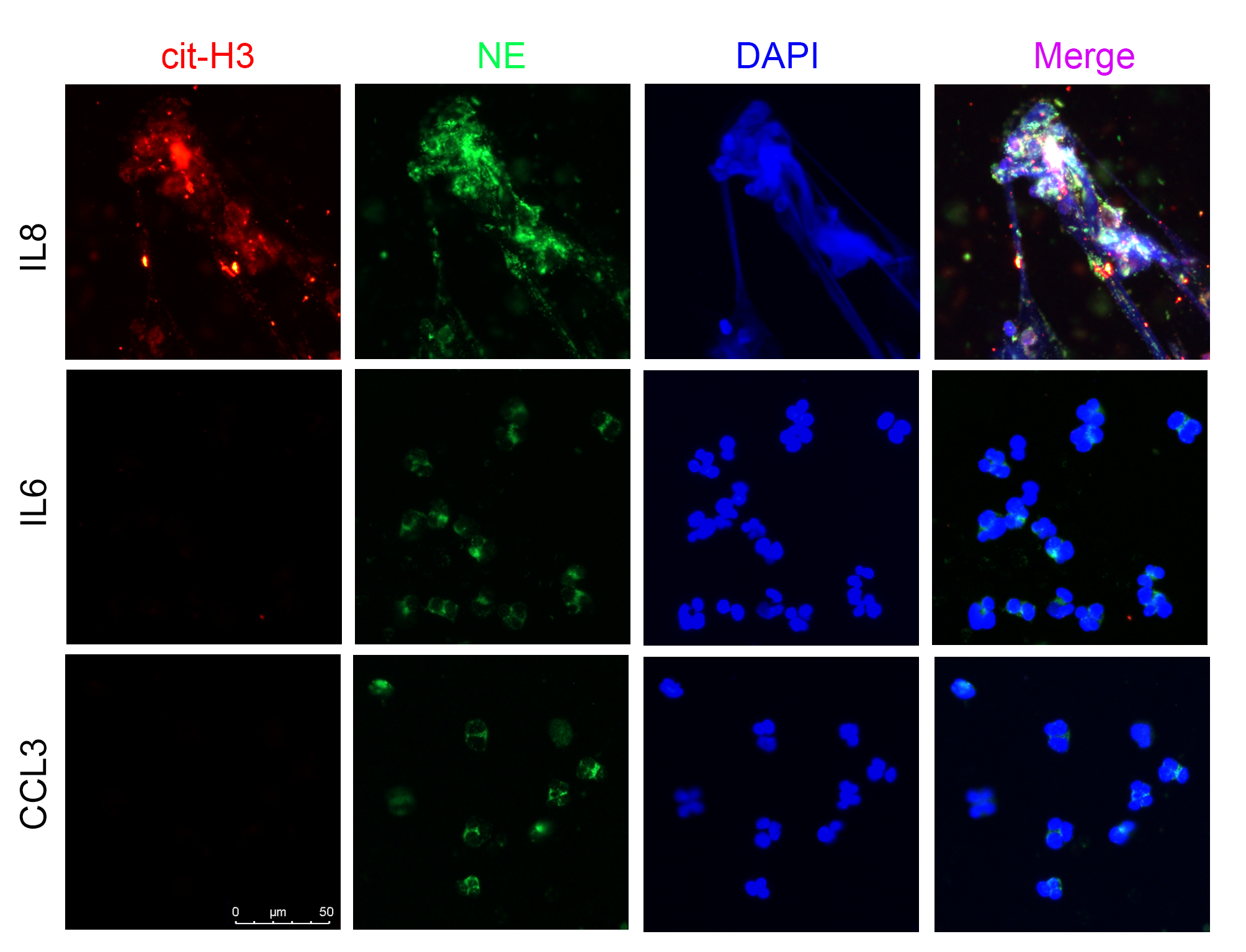

Supplement: Supplementary file 5 — Suppl.Fig. S4 [file 41419_2020_3286_MOESM5_ESM.tif]

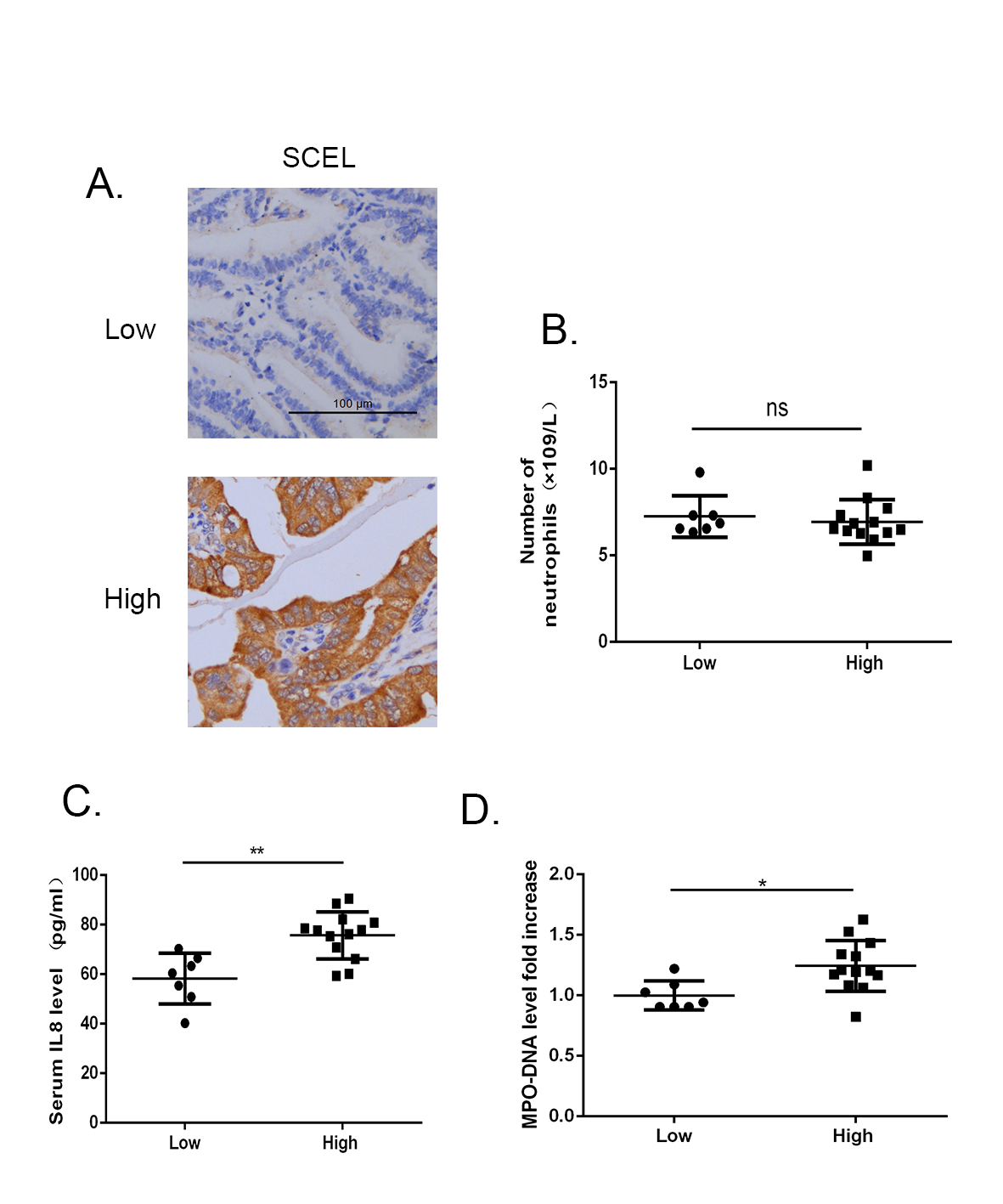

Supplement: Supplementary file 6 — Suppl.Fig. S6 [file 41419_2020_3286_MOESM6_ESM.tif]
