# Supplementary material for: Role of Sciellin in gallbladder cancer proliferation and formation of neutrophil extracellular traps
Source: Cell Death Dis. 2021 Jan 6;12(1):30. doi: 10.1038/s41419-020-03286-z (PMC7791032; doi:10.1038/s41419-020-03286-z)
Supplement: Supplementary file 7 — suppl.figure legend [file 41419_2020_3286_MOESM7_ESM.docx]

**Suppl. Fig. S1** The expression of SCEL in five gallbladder cancer cells

**Suppl. Fig. S2 The expression correlation of EGFR with SCEL.** The indicated GBC cells with SCEL downregulation were subjected to immunofluorescence analysis

**Suppl.Fig. S3 The SCEL promotes GBC cell proliferation through p-EGFR-PI3K-AKT pathway** A,The indicated GBC cells were incubated with or without Erlotinib/LY294002 and then subjected to Western blotting to detect the expression level of p-EGFR(Y1068), PI3Kα110，p-AKT(ser473) B, The indicated GBC cells were transfectd EV or SCEL plasmid and treated Erlotinibor LY294002 and CCK8 assay was profromed to determine its proliferation.*p<0.05;**p<0.001,***p<0.001

**Suppl.Fig. S4 Different cytokines induce neutrophil extracellar traps.** IL8,IL6,CCL3 add to neutrophil culture medium after 3.5hours and then representative immunofluorescent staining photographs of cit-histone3(red), NE（green）and DAPI(blud)are shown by fluorescence microscope.

**Suppl.Fig. S5** The indicated GBC cell lines incubation with or without IL8 neutralization and then cocultured with neutrophil to identity the neutrophil extracellar traps formation

**Suppl.Fig. S6** A, Representative IHC images of SCEL expression using human GBC tissue (n = 20) Bars, 100 µm, B, Level of IL8 concentration in the GBC patients’ plasma, C, The numbers of neutrophils in blood ，D, Level of MPO-DNA complexes in the GBC patients’ plasma
