# Supplementary material for: Role of Sciellin in gallbladder cancer proliferation and formation of neutrophil extracellular traps
Source: Cell Death Dis. 2021 Jan 6;12(1):30. doi: 10.1038/s41419-020-03286-z (PMC7791032; doi:10.1038/s41419-020-03286-z)
Supplement: Supplementary file 8 — suppl.Table1 [file 41419_2020_3286_MOESM8_ESM.docx]

| Antibody | Source | Catalog no |
| --- | --- | --- |
| anti-SCEL | ABCAM | Ab197087 |
| anti-p-EGFR | ABclonal | AP0301 |
| anti-EGFR | ABclonal | A11575 |
| anti-PI3Kα110 | CST | #4255 |
| anti-p-AKT(ser-473) | CST | #4060 |
| anti-pan-AKT(ser-473 | CST | #4658 |
| anti-FLAG | ABclonal | AE063 |
| anti-ACTB | ABclonal | AC026 |
| anti-MPO | ABclonal | AB1374 |
| anti-cit-H3 | NOVUS | NB-100-57135 |
| anti-NE | NOVUS | MAB9167 |

suppl.Table1 Antibody list
