# Supplementary material for: Role of Sciellin in gallbladder cancer proliferation and formation of neutrophil extracellular traps
Source: Cell Death Dis. 2021 Jan 6;12(1):30. doi: 10.1038/s41419-020-03286-z (PMC7791032; doi:10.1038/s41419-020-03286-z)
Supplement: Supplementary file 9 — suppl.Table2 [file 41419_2020_3286_MOESM9_ESM.docx]

SCEL-primer F: TCGGTACAGTTCTGATGACACT

R: AACATGGACATGCTCCTATTGG

EGFR-primer F: AGGCACGAGTAACAAGCTCAC

R: ATGAGGACATAACCAGCCACC
